# What Is the Optimal Cut-Off Point of the 10-Item Center for Epidemiologic Studies Depression Scale for Screening Depression Among Chinese Individuals Aged 45 and Over? An Exploration Using Latent Profile Analysis

**DOI:** 10.3389/fpsyt.2022.820777

**Published:** 2022-03-14

**Authors:** Hanlin Fu, Lulu Si, Ruixia Guo

**Affiliations:** Department of Obstetrics and Gynecology, The First Affiliated Hospital of Zhengzhou University, Zhengzhou, China

**Keywords:** cut-off point, 10-item Center for Epidemiologic Studies Depression Scale, latent profile analysis, receiver operating characteristic, China Health and Retirement Longitudinal Study

## Abstract

**Background:**

The main objective of the current study was to gain insight into the heterogeneity and profiles of depressive symptoms in Chinese individuals aged 45 and over and to determine the optimal cut-off point for the 10-item Center for Epidemiologic Studies Depression Scale (CES-D-10) to provide a reference for future practical application.

**Methods:**

The participants were 16,997 Chinese community-dwelling adults aged 45 years or older who completed survey interviews for the 2018 China Health and Retirement Longitudinal Study. The current study utilised latent profile analysis (LPA) to identify distinct profiles based on participants’ responses to CES-D-10 items, and receiver operating characteristic (ROC) curve analyses were applied to determine the optimal cut-off point for the CES-D-10 scale.

**Results:**

A three-profile solution was suggested as the optimum and included a “minimal depression” group (63.1%), “mild depression” group (23.4%) and “moderate-severe depression” group (13.5%); 36.9% (95% CI: 36.2 ∼ 37.6%) were considered at risk for probable depression. The “minimal depression” group was viewed as “non-cases,” and the remaining were viewed as “cases” that served as the reference standard for the ROC analysis, which obtained an AUC value of 97.8% (95% CI: 97.7–98.0%) and identified an optimal cut-off point of 10 (sensitivity:91.93%, specificity: 92.76%, and accuracy: 92.45).

**Conclusion:**

The identification of these distinct profiles underscores the heterogeneity in depressive symptoms among Chinese middle-aged and older adults. The CES-D-10 scale was demonstrated to have acceptable psychometric properties, with a cut-off point of 10 recommended for future research and practical application.

## Introduction

Depression is a common psychiatric disorder that can seriously threaten a person’s physical and mental health and wellbeing. According to the World Health Organization, more than 264 million individuals of all ages are afflicted by depression across the globe (1). As a major public health concern and social problem, depression causes a substantial global burden of morbidity and mortality. Estimates from the 2017 and 2019 Global Burden of Disease study highlight that depressive disorders contribute to 14.3% of all years lived with disability (YLDs) and 1.8% of all disability-adjusted life years (DALYs), which places these disorders among the three leading causes of YLDs and as the 13th leading causes of DALYs (1, 2). Moreover, a growing body of evidence supports depression being associated with higher risks of cardiovascular disease (3), hypertension (4), diabetes (5), cancer (6), and even suicide (7), further increasing the burden of the disease in high-income countries as well as in low- and middle-income countries. Therefore, effective and systematic screening of depressed individuals in the general population has been advocated because it is critical for timely diagnosis and intervention and monitoring disease prevalence, which can help to mitigate resource challenges and relieve the public health burden.

The identification of cases with depression strictly requires a clinical interview; because this process is time-consuming, burdensome and costly, it is unfeasible and unsuitable in large population-based epidemiological research. In the last few decades, several instruments have been developed to measure self-reported depressive symptoms in the general population. Arguably, the 10-item Center for Epidemiologic Studies Depression Scale (CES-D-10), proposed by Andresen et al. (8), is among one of the most widely used screening tools for assessing depression risk in population-based surveys and primary care settings. The CES-D-10 scale is a simplified version of the 20-item CES-D scale (9) that was generated from the original scale with the intent improving its clinical utility and reducing interviewee burden. Previous research comparing the CES-D-10 and the full scale has found high item–total correlations and comparable accuracy between them (8, 10, 11). The psychometric properties of the CES-D-10 have been evaluated in populations from various cultural backgrounds and with various clinical conditions, such as older adults in the United States (12), China (13), and Singapore (14), adolescents in Canada (15) and South Africa (16), HIV-positive people in Canada (10) and Colombia (17), psychiatric patients in the United States (18) and Japan (19), and multi-racial college students from 27 low- and middle-income countries (20), which all demonstrated satisfactory reliability and validity. Moreover, when compared with a formal psychiatric diagnosis or DSM-IV diagnosis of depression, the CES-D-10 at specific cut-offs produced acceptable sensitivity and specificity (19, 21), such that it exhibited adequate capacity to distinguish participants with and without depression. However, different optimal cut-off points of the CES-D-10 for detecting depression have been used and vary considerably from 5 to 16 (8, 18, 22–27). Such biases in the definition could lead to overestimation or underestimation of disease burden and inaccurate or even incorrect associations between depression and certain variables. Therefore, there is a need to clarify the optimal cut-off point for the CES-D-10 scale to provide a reference for future epidemiological screening in this field.

Clinical psychiatric interviews, serving as the gold standard for depression diagnosis, are commonly used to evaluate the performance of screening tools and estimate the optimal cut-off points. However, in the absence of an accurate reference standard, latent profile analysis (LPA) has been proposed to determine the disease status classification of individuals and thus to derive sensitivity and specificity for calculating cut-off points of assessment tools (28, 29). LPA is a person-centred statistical method that can identify unobserved heterogeneity in a population based on how they respond to continuously scored variables and group individuals with similar response patterns into homogenous subgroups (30–32). Therefore, individuals are homogenous within a subgroup but heterogeneous from different subsets. Although it is somewhat arbitrary to determine the number of latent profiles owing to a semi-subjective assessment of the model, the misclassification rates for participants produced by LPA are relatively low (33). Based on the results of LPA analysis, participants in the latent profile that represents the lowest risk of the disorder are considered “non-cases,” while the remaining participants are considered “cases.” The two groups obtained above are next used as the “gold standard.” A standard receiver operator characteristic (ROC) curve was then applied to determine the sensitivities and specificities of the screening instrument at different cut-scores against the reference standard. Multiple recent studies have applied LPA to explore the cut-off points and performance for several screening instruments (28, 34–36).

As with other low- and middle-income countries, depression is increasingly recognised as a major public health concern in China, particularly among elderly adults owing to rapid population ageing. The prevalence of depression among older Chinese adults varies from 23.6 to 43% (37–39). Therefore, there is an urgent need for depression screening and early intervention in this age group in China. The CES-D-10 has been validated to be appropriate for assessment of the elderly population in Chinese settings (13, 40). However, to the best of our knowledge, no domestic research has focused on the identification of the optimal cut-off point for the CES-D-10, which is imperative to accurately identify individuals with or without a risk of depression. Thus, the aims of this paper were (1) to examine the heterogeneity and profiles of depressive symptoms in Chinese individuals aged 45 and over by using LPA and (2) to determine the optimal cut-off point for the CES-D-10 to provide a reference for future empirical studies and practical applications.

## Materials and Methods

### Sample

This study utilised data from wave 4 (2018) of the China Health and Retirement Longitudinal Survey (CHARLS), which was officially released in September 2020. The CHARLS, an ongoing nationally representative survey of Chinese populations aged 45 years or older, is a cohort study conducted by the National School of Development at Peking University to facilitate the needs of scientific research on ageing-related issues (41). Based on a multi-stage stratified probability proportional-to-size (PPS) sampling method, baseline data collection was carried out *via* a face-to-face computer-assisted personal interview (CAPI) in 2011, and follow-up surveys were performed in 2013, 2015, and 2018. Further detailed descriptions of the survey are available elsewhere.

In the fourth-wave survey of the CHARLS, 19,816 respondents were interviewed from 150 counties of 28 provinces across China. The survey data could be freely and openly accessed at http://charls.pku.edu.cn/en. The inclusion criteria for the subjects were as follows: (1) age ≥ 45 years old; and (2) complete information on all relevant variables listed below. Based on these criteria, a total of 16,997 participants’ data were analysed in this study. Ethics approval for CHARLS data collection was granted by the Ethics Review Committee of Peking University (IRB 00001052–11015) and all participants provided informed consent.

### Measures

#### Depressive Symptoms

Depressive symptoms were measured using the CES-D-10, which comprised two parts: eight items on negative experiences (e.g., “I felt lonely” and “I felt depressed”) and two items on positive experiences (“I felt hopeful about the future” and “I was happy”). Each item was rated on a 4-point Likert scale: (1) rarely or none of the time (<1 day); (2) some or a little of the time (1–2 days); (3) occasionally or a moderate amount of the time (3–4 days); and (4) most or all of the time (5–7 days). Prior to the analysis, the eight negative items were scored from 0 (rarely or none of the time) to 3 (most or all of the time (5–7 days)), and the two positive items were reverse scored from 3 (rarely or none of the time) to 0 [most or all of the time (5–7 days)]. Thus, the overall score could range from 0 to 30, with higher scores indicating more severe depressive symptoms.

#### Other Relevant Information

Other relevant information collected in the study mainly included three aspects: sociodemographic characteristics, health-related behaviours and conditions. Sociodemographic information including age, gender (male, female), education level (primary school or below, middle school, high/vocational school, college degree or above), marital status (married/cohabitating, divorced/separated/widowed/never married), residential status (villages, cities/towns), and geographic region, was collected. Regarding geographic region, the twenty-eight provinces of China covered by the survey were classified into three regions according to their geographic location and economic level: eastern (10 provinces), central (8 provinces), and western (10 provinces). Health-related behaviours were assessed across three domains: drinking alcohol, smoking and night sleep duration. Information on smoking and drinking was collected by asking the respondents whether they had ever smoked (yes, no) and whether they consumed alcohol in the past year. Regarding drinking, possible answers were categorised as a dichotomous variable: yes (drink more than once a month/drink but less than once a month) or no (neither of these). Night sleep duration was ascertained by using the following question: “During the past month, how many hours of actual sleep did you get every night (average hours for one night)?” With respect to health status, three indicators were measured: disabilities, chronic disease and basic activities of daily living (BADLs). Chronic diseases were defined as having any of thirteen chronic conditions, including hypertension, dyslipidaemia, diabetes/high blood sugar, cancer/malignant tumour, chronic lung disease, liver disease, heart disease, stroke, kidney disease, stomach or other digestive diseases, memory-related disease, arthritis, rheumatism, or asthma. Disabilities were identified by asking whether the participants had at least one of the following conditions: physical disabilities, brain damage/intellectual disability, vision problems, hearing problems, or speech impediments. BADLs were evaluated by asking participants if they had difficulty with any of the following six activities: dressing, bathing or showering, eating, getting in or out of bed, using the toilet and controlling urination and defecation (42). The responses for each activity were dichotomised into yes (I have difficulty but can still do it/Yes, I have difficulty and need help/I cannot do it) or no (No, I don’t have any difficulty), and BADL disability was defined as having one or more of the six activities mentioned above.

### Statistical Analysis

First, descriptive statistics were applied to summarise the general characteristics of the participants. Continuous variables are reported as the mean ± standard deviation ($\bar{x}\pm s$), whereas categorical variables are presented as numbers and percentages.

Second, using the CES-D-10 items as indicators, LPA was performed through robust maximum likelihood estimation to identify distinct patterns of depressive symptoms among middle-aged and elderly Chinese individuals. A series of LPA models were fitted and compared to determine the best fitting model. As recommended by previous studies, the optimal model was selected based on several fit indices, including the Akaike information criterion (AIC), Bayesian information criterion (BIC), sample-size adjusted BIC (aBIC), bootstrap likelihood ratio test (BLRT), Lo–Mendell–Rubin test (LMRT), and entropy (43, 44). Generally, lower AIC, BIC, and aBIC values indicate a relatively better fit. However, it is sometimes difficult to identify an optimal model based on information criteria in that these indicators usually decrease along the increase in the number of extracted latent profiles. Therefore, the scree plot for the aBIC as well as its “elbow” point was visually inspected to confirm an appropriate number of clusters (28). Entropy, a standardised measure of how individuals are assigned to correct latent groups, was regarded as indicative of classification accuracy where an entropy value of 0.80 or higher represented adequate quality of classification (28, 44). Additionally, the high average latent profile probabilities for most likely latent profile membership were also examined, with values ≥ 0.9 indicating well-separated profiles. The LMRT and BLRT were employed to test the discrepancy between two models (i.e., k-class vs. k-1-class), and significant *P*-values provided support that the k-class was preferable (28, 44, 45). Taken together, the number of latent profiles was determined by a combination of the above fit criteria, rather than a single one. The logic and procedure for model selection are described in the following Section “Results.” Furthermore, Cohen’s *d* was calculated as a measure of effect size to elucidate the magnitude of the profile-level difference (46). Cohen’s *d*-values of 0.2, 0.5, or 0.8 indicate a small, medium or large effect sizes, respectively. On the basis of the optimal model, a 3-step approach that took into account the classification error was recommended to explore the effects of sample characteristics on profile memberships in the original measurement model (47).

Third, receiver operating characteristic (ROC) analysis was performed to determine the optimal cut-off value for the CES-D-10. The performance of the classifiers was evaluated by the area under the ROC curve (AUC), sensitivity, specificity, positive predictive value (PPV), negative predictive value (NPV), accuracy, and Youden’s index value (sensitivity + specificity – 1). Among those measures, the AUC was used as a measure of diagnostic accuracy such that an AUC value close to 1 indicated perfect diagnostic power. The optimal cut-off point was identified based on the maximum Youden’s index. All analyses in the study were completed with Mplus version 8.3 and R version 4.0.3. A two-sided *P*-value < 0.05 was considered statistically significant.

## Results

### Subjects Characteristics

Table 1 presents the general characteristics of the included participants. Of the 16,997 participants, 47.96% were male, and their mean age was 61.54 ± 9.44 years. The participants with primary school or below accounted for 56.8% of the sample, followed by middle school (22.58%), high/vocational school (10.99%), and college degree or above (2.07%). The majority of the participants (86.49%) were married or cohabiting, while the remaining participants (13.51%) were separated, divorced, widowed, or never married. A total of 71.54% of the participants resided in a village and 28.56% lived in a city or town. All participants were approximately evenly distributed across the eastern (33.29%), central (33.44%), and western (33.27%) regions of China. Regarding health status, approximately four-fifths of the study population had been diagnosed with at least one chronic disease, more than one-third suffered from at least one disability, and 17.17% reported having a BADL disability. The survey also found that nearly half (42.85%) of the respondents currently smoked or had ever smoked, and 34.94% had consumed alcohol in the previous year. The average night sleep duration for the sample was 6.20 ± 1.95 h. In addition, the mean score for depressive symptoms on the CES-D-10 was 8.71 ± 6.58.

**TABLE 1:** General characteristics of participants (*n* = 16997).

Variable	Total
Age, years ($\bar{x}\pm s$)	61.54 ± 9.44
**Gender (%)**	
Male	8151 (47.96)
Female	8846 (52.04)
**Education (%)**	
Primary school or below	10940 (64.36)
Middle school	3838 (22.58)
High/vocational school	1868 (10.99)
College degree or above	351 (2.07)
**Marital status (%)**	
Married/cohabitated	14701 (86.49)
Separated/divorced/widowed/never married	2296 (13.51)
**Residential area (%)**	
Village	12159 (71.54)
City/town	4838 (28.46)
**Geographic region (%)**	
Eastern region	5659 (33.29)
Central region	5683 (33.44)
Western region	5655 (33.27)
**Disabilities (%)**	
Yes	5882 (34.61)
No	11115 (65.39)
**Chronic disease (%)**	
Yes	13656 (80.34)
No	3341 (19.66)
**Smoking (%)**	
Yes	7283 (42.85)
No	9714 (57.15)
**Drinking (%)**	
Yes	5938 (34.94)
No	11059 (65.06)
**BADL**	
Yes	2919 (17.17)
No	14078 (82.83)
Depressive scores ($\bar{x}\pm s$)	8.71 ± 6.58
Sleep duration ($\bar{x}\pm s$)	6.20 ± 1.95

*BADL, basic activities of the daily living.*

### Latent Profile Analysis

**FIGURE 1:**
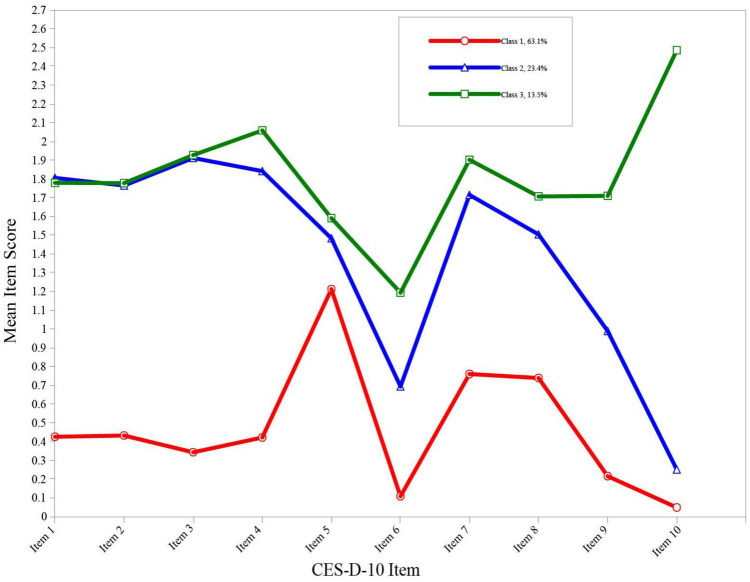
Scree plot of aBIC from LPA analysis. aBIC, adjusted Bayesian information criterion; LPA, latent profile analysis.

The LPA results showed that the models converged up to six profiles with no error warning. Therefore, only six LPA models were obtained. The fit indices for one- to six-profile solutions are presented in Table 2, and subsequently compared. First, we observed that the value of entropy in each model was above 0.90 with the exception of the two-profile solution (0.898), indicating that all models could provide high classification accuracy. Second, since the BLRT and LMRT results were significant for every model comparison, these two indicators were non-informative for current model selection. Third, as the number of profiles increased, the AIC, BIC and aBIC values continued to decrease across the six profiles. By visual inspection of the scree plot from aBIC, we found an “elbow” point at the three-profile solution, which indicated a considerably improved fit when the number of latent profiles increased from 2 to 3, but there were diminishing returns in model fit with another cluster added to the three-profile model (Figure 1). Taken together, the three-profile solution was therefore selected as the optimal model for the current sample.

**TABLE 2:** Model fit indices for one- to six-latent profile solutions and corresponding profile prevalence.

Model	k	AIC	BIC	aBIC	Entropy	LMR	BLRT	Profile prevalence
1C	20	507333.777	507488.592	507425.034	–	–	–	1
2C	31	468278.594	468518.559	468420.043	0.898	0.0000	0.0000	0.710/0.290
**3C^#^**	**42^#^**	**450841.173^#^**	**451166.286^#^**	**451032.813^#^**	**0.920^#^**	**0.0000^#^**	**0.0000^#^**	**0.631/0.234/0.135^#^**
4C	53	445237.999	445648.261	445479.830	0.928	0.0000	0.0000	0.065/0.237/0.628/0.070
5C	64	433192.110	433687.520	433484.132	0.943	0.0000	0.0000	0.078/0.595/0.062/0.057/0.207
6C	75	429886.904	430467.464	430229.119	0.923	0.0000	0.0000	0.062/0.078/0.486/0.194/0.122/0.057

*AIC, Akaike information criterion; BIC, Bayesian information criterion; aBIC, adjusted Bayesian information criterion; LMR, Lo–Mendell–Rubin test; BLRT, bootstrap likelihood ratio test. ^#^Selected as the optimal model.*

For this three-profile model, the entropy value (0.920) and the average latent profile probabilities for the most likely latent profile membership (0.968, 0.928, and 0.998, respectively) both demonstrated good distinction (Table 3). Profile 1, comprising 63.1% of the sample (*n* = 10728), was characterised by the lowest level of depressive symptoms (4.69 ± 3.17) and was labelled the “minimal depression” group. Profile 2, accounting for 23.4% of the sample (*n* = 3968), was characterised by an intermediate level of depressive symptoms (14.13 ± 3.84) and was labelled the “mild depression” group. Profile 3, consisting of 13.5% of the sample (*n* = 2301), was characterised by the highest level of depressive symptoms (18.10 ± 5.75) and was therefore labelled the “moderate-severe depression” group.

**TABLE 3:** Average latent profile probabilities for most likely latent profile membership (row) by latent profile (column).

Latent profile	Latent profile membership		
	1 (10728)	2 (3968)	3 (2301)
1	0.968	0.032	0.000
2	0.072	0.928	0.000
3	0.002	0.000	0.998

*The columns refer to latent profile, and the rows refer to most likely profile membership. Profile 1 = minimal depression; Profile 2 = mild depression; Profile 3 = moderate-severe depression.*

Table 4 displayed means, standard deviations, and Cohen’s *d* for the three profiles of CES-D10. There were significant differences regarding the overall mean scores on the CES-D-10 among the three groups (*P* < *0.05*). Pairwise comparison demonstrated that all differences among three profiles reached a significant level with large effect sizes (0.81–2.88).

**TABLE 4:** Means, standard deviations, and Cohen’s *d* for the three profiles of CES-D-10.

Profile 1		Profile 2		Profile 3		*d*_2–1_	*d*_3–1_	*d*_3–2_
M	SD	M	SD	M	SD			
4.69	3.17	14.13	3.84	18.10	5.75	2.68	2.88	0.81

*Profile 1 = minimal depression; Profile 2 = mild depression; Profile 3 = moderate-severe depression. M, Means; SD, standard deviation.*

**FIGURE 2:**
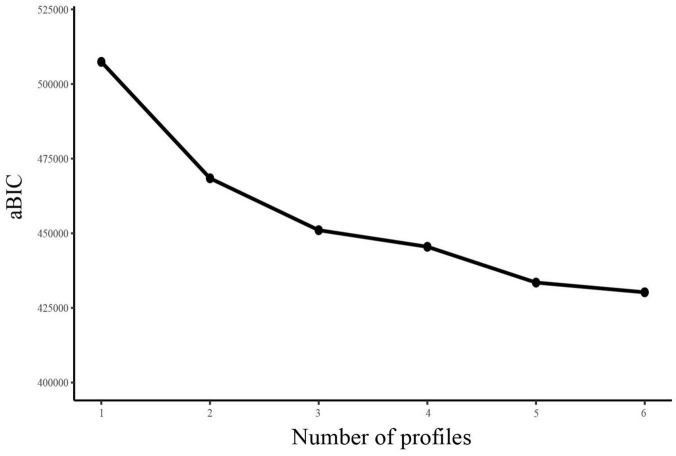
Three profiles of the best-fitting three-class pattern based on CES-D-10 items. CES-D-10, 10-item Center for Epidemiologic Studies Depression Scale.

Individuals were considered probably depressed if they were classified in the “mild depression” group or “moderate-severe depression” group; therefore, the prevalence of depression among the study participants was 36.9% (95% CI: 36.2∼37.6%). The distribution of the mean scores for each CES-D-10 item for the three profiles is presented in Figure 2.

To identify predictors of profile membership, a 3-step approach nested within in LPA was conducted with the “minimal depression” group as a reference. The results showed that age, gender, education, marital status, residential status, geographic region, disabilities, chronic disease, smoking, drinking, sleep duration, and BADL disability were significantly associated with latent profile memberships (*P* < 0.05) (Supplementary Table 1). Specifically, compared to those in the “minimal depression” group, participants belonging to the “mild depression” group and “moderate-severe depression” group were more likely to be young, female, have a low education level, reside in rural areas and central/western regions, and report having disabilities, chronic diseases, BADL disabilities and short sleep duration. Moreover, the participants who were separated/divorced/widowed/never married, smoked or consumed alcohol in the past year, were more likely to be present in the “moderate-severe depression” group.

### Receiver Operating Characteristic Analysis

**FIGURE 3:**
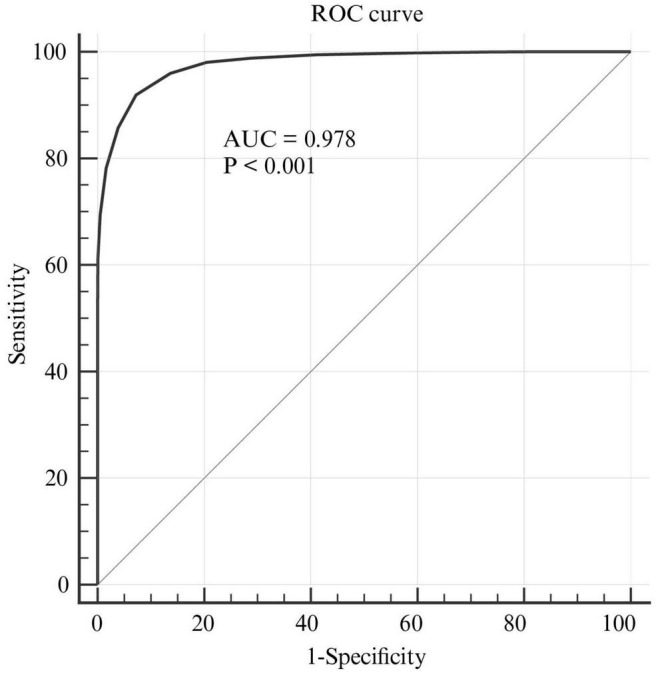
The ROC curve of the CES-D-10 for screening depression. ROC, receiver operating characteristic; CES-D-10, 10-item Center for Epidemiologic Studies Depression Scale.

According to the LPA results, individuals assigned to the “minimal depression” group were defined as “non-cases” (i.e., no depression), while those in the “mild depression” or “moderate-severe depression” group were defined as “cases” (i.e., probable depression). Using this binary outcome, the ROC curve was plotted for the CES-D-10, with an AUC value of 97.8% (95%: 97.7–98.0%), indicating a good predictive capacity for depression (Figure 3). Table 5 presents the diagnostic indices for potential cut-off points from 7 to 13. The optimal cut-off value of CES-D-10 was ≥10, which corresponded to the maximum Youden index (Youden index = 0.847). In this case, the sensitivity, specificity, PPV, NPV, and accuracy were 91.93, 92.76, 88.12, 95.16, and 92.45%, respectively.

**TABLE 5:** Criterion values and coordinates of ROC Curve for depression.

Cut-off point	Sensitivity	Specificity	PPV	NPV	Accuracy	Youden index
7	98.82	71.24	66.75	99.04	81.41	70.06
8	98.04	79.52	73.67	98.58	86.35	77.56
9	95.95	86.34	80.41	97.33	89.89	82.29
**10^#^**	**91.93^#^**	**92.76^#^**	**88.12^#^**	**95.16^#^**	**92.45^#^**	**84.69^#^**
11	85.76	96.12	92.82	92.03	92.30	81.88
12	78.19	98.38	96.57	88.53	90.93	76.57
13	69.37	99.52	98.82	84.76	88.40	68.89

*ROC, receiver operating characteristic; PPV, positive predictive value; NPV, negative predictive value. ^#^Selected as the optimal cut-off value.*

## Discussion

As far as we know, this is one of the few studies using LPA to identify distinct classes of depressive symptoms in a Chinese community sample of middle-aged and older adults (45+). In the process of model fitting, a total of six LPA models were tested, and the optimal model was determined through comparison of several fit indices and visual inspection of the scree plot. Eventually, a three-profile solution was selected to be the best fit to the current data, and the average latent profile probabilities for the most likely latent profile membership demonstrated strong discrimination, underscoring the presence of heterogeneity in depressive symptoms among this population. The values of Cohen’s *d* > 0.8 further confirmed the accuracy of the classification. The result was similar to a previous study that was also conducted in the Chinese elderly individuals (60+) but used the Zung self-rating depression scale (48). Based on the scoring pattern of the CES-D-10 and the mean score in each profile, the three profiles were labelled the “minimal depression” group (63.1%), “mild depression” group (23.4%) and “moderate-severe depression” group (13.5%). Individuals with probable depression were defined as those who belonged to the last two groups, and therefore the overall prevalence of depression among older adults was estimated to be 36.9%, which was roughly in agreement with results from previous studies (37, 39).

The “minimal depression” subgroup was characterised by the lowest mean scores across all CES-D-10 items, with an overall mean score of 4.69 ± 3.17 for the scale. Interestingly, there were three peak values in item 5, item 7, and item 8 (see Figure 2). Item 5 (i.e., I felt hopeful about the future) and item 8 (i.e., I was happy) are the only two “positive affect” items that are reverse coded on the CES-D-10. The simultaneous appearance of two peaks corresponding to the above two items did not seem to be a pure coincidence and definitive elevations. A prior study by Saracino et al. (49) suggested that a certain proportion of participants may be subject to “patterned responses” on the CES-D. There was therefore a strong likelihood that they intended to indicate “no symptom” on all items but accidentally endorsed these two reverse-coded items in a discordant manner due to their inattention. Swain et al. (50) found that the average error of response to reverse-coded Likert items was approximately 20%. Some studies have questioned the utility and reliability of reverse-coded items in questionnaire (49, 51–53). With regard to peak value for item 7 (i.e., my sleep was restless), a possible explanation for this might be that sleep complaints are common among older adults, which has been indicated in a variety of recent studies (54–58). For the “mild depression” group and “moderate-severe depression” group, the mean scores across all indicators were higher and nearly consistent across the two groups with the exception of item 10 (i.e., I could not get “going”), which showed a substantial distinction. Literally, the item 10 can be understood as an indication of suicidal ideation in the Chinese cultural context, which to some extent reflected a severely depressed mood and served as the basis for defining the two groups in the current study. Additionally, the overall mean CES-D-10 score in the “moderate-severe depression” group (18.10 ± 5.75) was significantly higher than that in the “mild depression” group (14.13 ± 3.84), further illustrating the heterogeneity between subgroups. It was noteworthy that item 6 (i.e., I felt fearful) corresponded to the trough value within each profile. There was a study in a sample of English- and French-speaking Canadians suggesting that differential item functioning in cross-linguistic comparisons for item 6 was observed, which may be attributed to translational or cultural differences (59). Accordingly, we surmised that the above phenomenon in this study may also be related to translation and culture.

The cut-off score for CES-D-10 is usually used as a guideline for further examination. Our study explored the cut-off threshold for the CES-D-10 by using a combination of LPA and ROC analysis, which contributed to defining the accuracy of the CES-D-10 in case identification. Based on the results, a CES-D-10 value of 10 was found to offer optimal discriminatory power in detecting individuals with or without a risk of depression in the Chinese population aged 45 years or older, with acceptable sensitivity, specificity, PPV, NPV, accuracy, and AUC values of 91.93, 92.76, 88.12, 95.16, 92.45, and 97.8%, respectively. Our findings were consistent with the cut-off value recommended by Andresen et al. (8) and Boey (60) but were not identical to studies that were also conducted with a Chinese sample by Cheng et al. (23, 27). This discrepancy could be attributed to differences in reference standards, clinical settings, concomitant diseases, cultures and sample sizes. For instance, Andresen et al. (8) used the conventional threshold of 16 for the CES-D-20 as the criterion to assess the CES-D-10 in a sample of American older adults. However, a recent meta-analysis by Vilagut et al. (61) showed that the CES-D-20 should not be used as an isolated diagnostic measure of depression, and the threshold value of 16 for this scale was not recommended in detecting depression in the general population. In another study, Cheng et al. (27) evaluated the diagnostic performance and threshold of the CES-D-10 by focusing on a small sample of elderly people with dementia in China. Unlike previous studies using validated screening tools or clinical interviews as the gold standard to determine disease status classification, we categorised the targeted populations into a “cases” group (i.e., probable depression) and “non-cases” group (i.e., no depression) based on the LPA results, which is thought to provide valid estimates of accuracy and is usually applied in diagnostic studies. Given that the CES-D-10 is a screening tool rather than a diagnostic tool, we recommended a cut-off value of 10 for CES-D-10 use in future population epidemiological studies.

Strengths of the study included the use of a large nationally representative sample, which potentially increased the accuracy of the results and contributed greater confidence in the generalizability of findings. Moreover, findings from this study extended the extant literature on the application of LPA in elucidating heterogeneity and profiles of depressive symptom presentation and deriving cut-off points for screening tools. However, there were several limitations to consider. First, although several objective indices, including AIC, BIC, aBIC, BLRT, LMRT, and entropy, were informative, they were inadequate to confirm the best fitting model. The three-profile solution was finally determined by a semi-subjective assessment, i.e., visual inspection of the scree plot of aBIC, which may have reduced the credibility and trustworthiness of the results. Therefore, the current findings require replication. Second, it should be emphasised that LPA is explorative in nature and assigns individuals to their respective profiles based on posterior probabilities, which may yield misclassification when the probability of individuals being distributed into two groups is roughly equal. However, our results demonstrated excellent separation. Third, the optimal cut-off for the CES-D was selected by using a sophisticated analytic method but not a formal clinical diagnosis, and the reliability of the result needs to be verified in future more extensive studies. In addition, a total of 16,997 out of 19,816 individual cases were included in the final analysis. Nevertheless, there were significant differences in sample demographics between the included cases and excluded cases, which may have led to biased results. Hence, caution is warranted when interpreting the results.

In summary, our study provided strong evidence supporting the heterogeneity in depressive symptoms among Chinese middle-aged and older adults. The CES-D-10 scale was demonstrated to have acceptable psychometric properties for initially screening depression in epidemiological surveys, and a cut-off point of 10 for CES-D-10 was recommended for future research and practical application.

## Data Availability Statement

The raw data supporting the conclusions of this article will be made available by the authors, without undue reservation.

## Ethics Statement

The studies involving human participants were reviewed and approved by the Ethics Review Committee of Peking University (IRB 00001052-11015). The patients/participants provided their written informed consent to participate in this study.

## Author Contributions

HF and RG contributed to the conception and design of the study. HF performed the statistical analysis and wrote the first draft of the manuscript. LS and RG reviewed the manuscript. All authors have read and approved the final version of the manuscript.

## Conflict of Interest

The authors declare that the research was conducted in the absence of any commercial or financial relationships that could be construed as a potential conflict of interest.

## Publisher’s Note

All claims expressed in this article are solely those of the authors and do not necessarily represent those of their affiliated organizations, or those of the publisher, the editors and the reviewers. Any product that may be evaluated in this article, or claim that may be made by its manufacturer, is not guaranteed or endorsed by the publisher.
