# Supplementary material for: What Is the Optimal Cut-Off Point of the 10-Item Center for Epidemiologic Studies Depression Scale for Screening Depression Among Chinese Individuals Aged 45 and Over? An Exploration Using Latent Profile Analysis
Source: Front Psychiatry. 2022 Mar 14;13:820777. doi: 10.3389/fpsyt.2022.820777 (PMC8963942; doi:10.3389/fpsyt.2022.820777)
Supplement: Supplementary file 1 [file Table_1.DOCX]

Supplementary Material

**Table S1. Sociodemographic characteristics, health-related behaviors and conditions as predictors of class membership.**

| Variables | Low risk | | | High risk | | |
| --- | --- | --- | --- | --- | --- | --- |
|  | *B* | *Exp(B)(95% CI)* | *P* | *B* | *Exp(B)(95% CI)* | *P* |
| Age(continuous) | -0.022 | 0.98(0.97-0.98) | <0.001^*^ | -0.018 | 0.98(0.98-0.99) | <0.001^*^ |
| Gender(male=ref) | 0.497 | 1.64(1.42-1.90) | <0.001^*^ | 0.492 | 1.64(1.39-1.92) | <0.001^*^ |
| Education (primary school or below=ref) | -0.196 | 0.82(0.77-0.88) | <0.001^*^ | -0.514 | 0.60(0.55-0.65) | <0.001^*^ |
| Marital status (married/cohabiting=ref) | 0.116 | 1.12(0.98-1.29) | 0.091 | 0.445 | 1.56(1.36-1.80) | <0.001^*^ |
| Residential area(rural=ref) | -0.354 | 0.70(0.63-0.78) | <0.001^*^ | -0.561 | 0.57(0.50-0.65) | <0.001^*^ |
| Geographic region(eastern=ref) | 0.253 | 1.29(1.22-1.36) | <0.001^*^ | 0.332 | 1.39(1.31-1.48) | <0.001^*^ |
| Disabilities(no=ref) | 0.430 | 1.54(1.40-1.69) | <0.001^*^ | 0.610 | 1.84(1.66-2.05) | <0.001^*^ |
| Chronic disease(no=ref) | 0.573 | 1.77(1.57-2.01) | <0.001^*^ | 0.446 | 1.56(1.35-1.81) | <0.001^*^ |
| Smoking(no=ref) | 0.042 | 1.04(0.91-1.20) | 0.552 | 0.169 | 1.18(1.01-1.39) | 0.035^*^ |
| Drinking(no=ref) | -0.102 | 0.90(0.81-1.00) | 0.054 | -0.318 | 0.72(0.64-0.82) | <0.001^*^ |
| Sleep duration(continuous) | -0.165 | 0.85(0.83-0.87) | <0.001^*^ | -0.187 | 0.83(0.81-0.85) | <0.001^*^ |
| BADL disability(no=ref) | 1.001 | 2.72(2.41-3.07) | <0.001^*^ | 1.326 | 3.77(3.33-4.26) | <0.001^*^ |

Note: ^*^ *P*<0.05

CI= confidence interval, BADL= basic activities of the daily living
